# Incidental finding of carcinoid tumor on Meckel’s diverticulum: case report and literature review, should prophylactic resection be recommended?

**DOI:** 10.1186/1477-7819-12-144

**Published:** 2014-05-08

**Authors:** Daniela Caracappa, Nino Gullà, Francesco Lombardo, Gloria Burini, Elisa Castellani, Carlo Boselli, Alessandro Gemini, Maria Federica Burattini, Piero Covarelli, Giuseppe Noya

**Affiliations:** 1 General and Oncologic Surgery Unit, Department of Surgical, Radiological and Odontostomatological Sciences, University of Perugia, Perugia, Italy; 2 General Oncologic Surgery, Papardo General Hospital, Messina, Italy

**Keywords:** Meckel’s diverticulum, Carcinoid, Surgery

## Abstract

Meckel’s diverticulum (MD) is the most common congenital anomaly of the gastrointestinal tract and is caused by incomplete obliteration of the vitelline duct during intrauterine life. MD affects less than 2% of the population. In most cases, MD is asymptomatic and the estimated average complication risk of MD carriers, which is inversely proportional to age, ranges between 2% and 4%. The most common MD-related complications are gastrointestinal bleeding, intestinal obstruction and acute phlogosis. Excision is mandatory in the case of symptomatic diverticula regardless of age, while surgical treatment for asymptomatic diverticula remains controversial. According to the majority of studies, the incidental finding of MD in children is an indication for surgical resection, while the management of adults is not yet unanimous. In this case report, we describe the prophylactic resection of an incidentally detected MD, which led to the removal of an occult mucosal carcinoid tumor. In literature, the association of MD and carcinoid tumor is reported as a rare finding. Even though the strategy for adult patients of an incidental finding of MD during surgery performed for other reasons divides the experts, we recommend prophylactic excision in order to avoid any further risk.

## Background

Meckel’s diverticulum (MD) is the most common congenital anomaly of the gastrointestinal tract and is caused by incomplete obliteration of the vitelline duct during intrauterine life. MD has an autoptic prevalence of 2% in the general population and an average rate of complications of between 2% and 4% [1]. The incidence of complications is inversely proportional to age, and is virtually nonexistent for adults over 70 years of age. Therefore, the majority of MD cases remain asymptomatic for life, and symptomatic cases occur almost exclusively in the earliest years of life. For these reasons, prophylactic excision is usually desirable for children, but remains controversial in the case of incidental diagnosis in asymptomatic adults. Current literature does not provide definitive evidence of the most appropriate treatment.

We present the case of a patient who underwent exploratory laparotomy for an acute abdomen due to intestinal perforation on a sigmoid diverticulum. During the operation we proceeded to the resection of an incidentally detected MD. The subsequent histological report showed a submucosal carcinoid tumor in the MD.

## Case presentation

### Clinical history

A 38-year-old male presented to the emergency room at Santa Maria della Misericordia Perugia, Italy, with a 12-hour history of abdominal pain associated with fever (38.2°C). The patient reported the acute onset of abdominal cramps mainly localized in the hypogastrium and left iliac fossa; neither alterations of bowel transit nor nausea and vomiting were reported. The patient’s personal and family history were negative for neoplastic disease, and pathological anamnesis revealed untreated allergic asthma and hypertension. Clinical examination revealed diffuse abdominal tenderness elicited on deep palpation of the lower quadrants and positive Blumberg sign. Blood tests showed neutrophil leukocytosis (11,060 white blood cell count, 81% neutrophils). Abdominal ultrasonography was normal, while X-ray showed air-fluid levels of the small bowel on the right flank.

A few hours after admission, the patient’s clinical conditions suddenly worsened with exacerbation of the abdominal pain. The patient consequently underwent emergency diagnostic laparoscopy. The intraoperative finding was a peritoneal purulent fluid collection with a large sigmoidal abscess, due to a perforated diverticulitis on the mesenteric bowel side. Laparotomic conversion was requested by the septic condition and resection of the sigmoid colon with prophylactic appendectomy was performed. Careful exploration of the abdominal content revealed MD, located approximately 50 cm above the ileocaecal valve, which was also prophylactically removed. Finally, a ghost ileostomy was created by collecting (without externalizing) the ileum to the abdominal wall, approximately 70 cm above the ileocaecal valve.

The pathologic examination of the specimen showed sigmoid diverticular disease, complicated by acute diverticulitis and fibrinopurulent peritonitis, scleroatrophic appendix and the presence of reactive mesothelial cells in the peritoneal fluid, in the absence of atypia. A carcinoid tumor was found in the submucosa of the MD, locally invading the mucosa but not the muscular layer on a ground of fibrinopurulent serositis.

**Figure 1:**
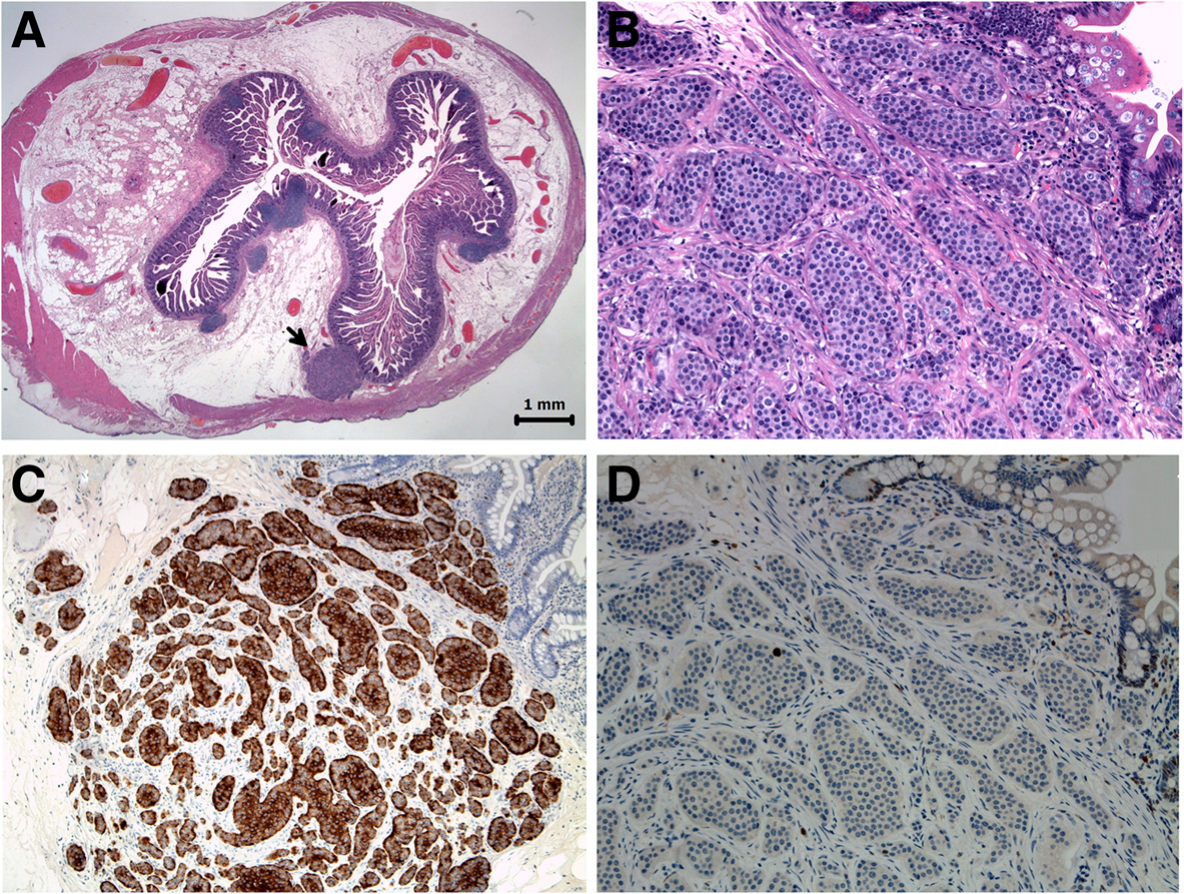
**Coronal section of the diverticular body. (A)** Submucosal nodule (arrow). H & E, original magnification 125×. **(B)** Neoplastic proliferation, with monomorphic cells organized into nests. H & E, original magnification 200×. **(C)** Intense and diffuse immunohistochemical positivity for chromogranin. Original magnification 100×. **(D)** Ki-67 (MIB-1) proliferation index of less than 1%. Original magnification 200×. H & E, hematoxylin and eosin.

This well-differentiated neuroendocrine tumor (measuring 1.25 mm on the histological section) was located at the level of the diverticular body. Immunohistochemistry was performed, revealing chromogranin positive, synaptophysin positive and a Ki-67 (MIB-1) proliferation index of less than 1% (Figure 1). According to the diameter of the tumor, simple diverticulectomy without bowel resection was considered adequate treatment.

The postoperative course was regular and the patient was discharged in good clinical condition on the 12th postoperative day. A computed tomography (CT) scan was subsequently performed and excluded metastatic dissemination. To date, the follow-up of the patient has been 3 years, and there has been no evidence of recurrence or metastatic disease.

### Discussion and literature review

MD is the most common congenital disease of the gastrointestinal tract, affecting approximately 2% of the population [1,2]. *Although described in 1598* by Fabricus Hildanus *as an anatomic varia*nt, in 1809, Johann Meckel was the *first* to *produce a detailed description on the subject.*

MD is the most common true diverticulum of the gastrointestinal tract. It is localized approximately 45 to 60 cm proximal to the ileocaecal valve on the antimesenteric side, on the projection of the terminal branch of the superior mesenteric artery (SMA), which represents the rotational axis of the fetal gut [3]. With regards to pathogenesis, MD is the result of incomplete obliteration of the vitelline duct between the 5th and 8th week of gestation [4], which may also rarely evolve as a fistula or a fibrous band [5]. MD may contain areas of ectopic mucosa, mainly of gastric type, or be the site of development for various proliferative lesions, including benign tumors such as leiomyomas and lipomas, malignancies such as malignant sarcomas and adenocarcinomas, or more commonly carcinoid tumors [6].

In most cases, MD is not symptomatic and diagnosis is accidental during a laparotomy or laparoscopy performed for other reasons (as in our case), or during radiographic study of the small bowel, but most frequently it is an autoptic finding [5]. The estimated average complication risk of patients with MD ranges between 2% and 4% [1,2], and appears to be inversely proportional to age: 4 to 5% under 2 years, 1% at 40 years and almost 0% at 70 years [7].

The most common complications of MD include intestinal obstruction (22 to 50%) and gastrointestinal bleeding (11.8%), and is often related to the presence of ectopic mucosa and inflammatory complications (20%). *Less common presentations are* associated with *Littré*’s *hernia (anecdotal), fistula (1.7*%*) or as* a *consequence of neoplastic degeneration (3.2*%*)*.

MD-associated tumors occur in approximately one-third of carcinoid cases (33%) [8], and *other histological types include adenocarcinomas, pancreatic carcinomas, intraductal papillary mucinous adenomas of* the *ectopic pancreas, gastrointestinal stromal tumors (GISTs), leiomyosarcomas, lymphomas, lipomas, adenomyomas and villous adenomas*. Carcinoid neuroendocrine tumors originate from enterochromaffin cells, which are probably originally located in the neural crest, and represent the most common primary tumor of the small intestine.

*In 1907, Oberdorfer* coined the term carcinoid to *describe a type of neoplasm that,* considering *the benign characteristics,* could be *distinguished from cancer.* In 1914, *Gosset and Masson detected the affinity of carcinoid cells for silver salts.* Carcinoids can theoretically occur in any anatomical region, but are most commonly found in the appendix, with the ileum being the second most affected site, usually in its last 60 cm. *These tumors can secrete various hormon*e*s, the most important of which are serotonin and substance P*. They may have malignant behavior but usually show a low aggressiveness, being asymptomatic in 70 to 80% of cases [9]. Symptoms of intestinal carcinoid tumors can be periodic abdominal pain, gastrointestinal bleeding and obstruction, or by the typical carcinoid syndrome (10 to 20%) with acute episodes of skin flushing, diarrhea, asthma attacks, hepatomegaly and development of cardiac lesions. *Carcinoid syndrome, supported by serotonin secretion, occurs in 45*% *of patients with massive liver metastasis and in 10* to *20*% *of* patients *affected by carcinoid of the MD.* Because of the non-specificity of symptoms, especially in the early phase, the average time between the onset of symptoms and diagnosis varies from 2 to 20 years [7]. Therefore, half of patients present a disseminated disease at the time of diagnosis [10].

Since both MD and carcinoid tumors are rare clinical entities, the occurrence of a carcinoid tumor on a MD is even more uncommon. Considering the limited dimension of the MD, it should be noted that it has the highest incidence of carcinoid transformation per cm^2^ of mucosal surface [6]. *Modlin* et al. [11]*reported that a*pproximately *0.48 to 0.74*% *of all carcinoids occur in the MD*.

The association between carcinoid tumors and MD seems, in fact, validated by a common embryological origin, arising from incorrect interactions between the neural crest and endoderm [12]. The average age of appearance of a carcinoid on a MD is 55 years, with an incidence 2.5 times higher in men than women [6]. Until 1988, 52 cases of carcinoid in MD had been described [13], and in 1997 a review identified 111 cases [14]. Currently, the Surveillance, Epidemiology, and End Results (SEER) Program of the National Cancer Institute, the authoritative source of information on incidence and survival of cancer in the US, has reported 121 cases [15].

Carcinoids localized in the appendix or in the colon usually have a lower aggressive behavior than those with bronchial and small bowel origin. *As demonstrated by Moyan*[16]*, MD*-*associated carcinoids have a similar immunophenotype of small bowel carcinoids and consequently a comparable biological behavior*. The clinical presentation is closely related to the disease stage: lesions smaller than 10 mm with intact muscle layers are rarely symptomatic, whereas those with more aggressive local characteristics are frequently associated with local and systemic signs and symptoms.

According to Moertel et al., carcinoid tumors smaller than 1 cm have an incidence of 2% of metastasis, whereas lesions with a size between 1 and 2 cm metastasize in 50% of cases, and those larger than 2 cm metastasize in 80% of cases [17]. *Much higher rates have been detected by Thompson*[18]*,* who demonstrated *an incidence of metastas*i*s of 18*% *for lesions smaller than 1 cm and 85*% *for* lesions *between 1 and 2 cm. The liver is the most commonly affected organ, with a 5-year survival of* approximately *30*% *in patients with hepatic metastases; lung and bone metastases are less frequent. Metastases are twice* as *common in women than men,* most likely *because of hormonal factors*. According to their ability to early metastasize, carcinoid tumors of MD should be considered as relatively aggressive. Therefore, according to some published studies, resection of the adjacent ileal segment and corresponding mesentery is recommended for tumors larger than 5 mm [6].

*In more than 70*% *of cases, carcinoids* originate at the distal extremity of the MD [7]. In our case, however, the tumor was localized in the middle third of the diverticulum.

The excision of MD is of course required in the case of symptoms, regardless of age, while for asymptomatic diverticula the most appropriate treatment to adopt is controversial. *According to the majority of* published studies*, the* incidental *finding of a MD in children is an indication* for *surgical resection,* while many others advise prophylactic excision of the MD in pediatric age, especially for young infants; however, the strategy to be followed in the case of MD in adults divides the experts [5-19].

Despite the availability of many publications on the management of an incidental finding of MD, *with more than* 600 *publications in the last ten years*, most are case reports and prospective or randomized studies, which can hardly be realized because of the rarity of the condition. *Many centers report their own experience* and *often disagree on epidemiology, but especially on surgical indications;* and, *to date, the strategy to be followed in* the *case of MD in adults* is not yet unanimous*.* In 1976, Soltero and Bill *collected 202 cases of complicated MD* that *under*went *emergency surgical treatment*[20]*. The*y calculated that the risk of developing complications in MD carriers was 4.2% at birth, with a progressive decrease to zero with older age. Considering that literature-reported mortality was 6 to 7% for complicated MD surgery compared to zero for elective diverticulectomy, and that associated morbidity was 11.1% and 8.9%, respectively, Soltero and Bill argued that surgical resection at birth (when the incidence of complications is greater) in 400 patients could have saved one life (mortality 6%), but would have been responsible for 36 cases of associated morbidity (morbidity 8.9%). In adulthood, with decreasing risk of complications, 800 prophylactic resections would be necessary to prevent one death. For this reason, they stated that in the absence of specific risk factors, the high risk of postoperative complications together with the low incidence of diverticulum-related complications does not justify the removal of an incidentally diagnosed MD (Table 1).

**Table 1:** Comparison of complication rates between prophylactic and therapeutic resection of MD

**Study**	**Complication rate of MD carriers**	**Complication rate of prophylactic diverticulectomy**	**Complication rate of diverticulectomy in symptomatic MD**	**Prophylactic resection?**
**Soltero** and **Bill**[19]	4.2% (birth) to 0% (adults)	8.9%	11.1%	No
**Cullen** et al. [20]	6.4%	2%	12%	Yes
**Zani** et al. [21]	1.3%	5.3%	-	No

MD, Meckel’s diverticulum.

In contrast, Cullen et al., *in a review published in 1994,* supported prophylactic diverticulectomy [21]. A higher lifetime risk of complications was calculated as 6.4%, *making prophylactic resection advisable before the age of 80* years*. Diverticulectomies performed for MD complications ca*rried *an operative mortality and morbidity of 2*% *and 12*%*, with a cumulative risk of long-term postoperative complications of 7*%*,* and an operative mortality and morbidity for incidental diverticulectomies of 1 to 2% and 2%, respectively. They concluded that prophylactic surgical excision of MD is indicated at any age, especially before 80 years of age (Table 1).

A more recent review by Zani et al. focused on the differences in terms of early and late complications in *2*,*975* incidentally detected MD cases, *comparing* patients *treated conservatively with those who underwent resection*[22]*.* The result was a significantly higher incidence of complications in patients undergoing prophylactic diverticulectomy (5.3%), compared to untreated MD (1.3%). *According to this review, the number of prophylactic diverticulectomies to perform in order to prevent one death* wa*s estimated to be 758 in childhood, 771 between 45 and 65 years* of age*, 911 between 65 and 75 years* of age, *and 1*,*111 in older patients.* Therefore, Zani et al. supported the theory of a conservative approach (Table 1).

Park et al. instead proposed a more accurate selection of MD carriers, with the aim of only submitting patients to surgery with a higher risk of complications [23]. Taking into account the frequency of some characteristics associated to symptomatic diverticula, such as age, gender, length and presence of ectopic tissue, Park et al. recommended the resection of MD in males, in patients younger than 50 years, in the presence of a diverticulum longer than 2 cm and with visible anatomical changes. According to this theory, the simultaneous presence of all four features would be associated with an expected complication rate of 70%, and the finding of three, two or one of the aforementioned criteria would correspond to a risk of 42%, 25% and 17%, respectively.

It is important to note that most of the available data refer to retrospective studies, in which patients had undergone laparotomic diverticulectomies. The advent of laparoscopy, which allows a comprehensive exploration of the abdominal cavity with a minimally invasive technique, while enabling the tangential resection with an endostapler in a quick and safe manner, could modify the indications for surgery [4].

A recent review by Thirunavukarasu et al. examined the controversy of elective resection of MD, focusing on the relative risk of malignant transformation [15]. They *analyzed epidemiology, incidence, stage at first diagnosis and survival in 163* cases *of MD* carcinoids *and 6*,*214* cases *originat*ing *from* the *ileum.* They argued that, given the low but increasing incidence of MD malignant transformation (1.44 per 10 million inhabitants) and its enhancement with age, together with an estimated risk of MD transformation of 70 times higher than all other ileal locations, incidental MD is best treated with resection. Our own experience confirms this strategy, considering that the excision of an incidentally found MD resulted in early detection of a carcinoid tumor with subsequent prevention of its spread.

Obviously, once a carcinoid on MD is diagnosed, treatment should be adapted according to disease stage. Simple MD excision is considered adequate by most of the studies in the case of lesions of less than 10 mm in size [24,25], while according to others it is sufficient only for those smaller than 5 mm [6]. For larger lesions, resection of the ileal tract and the corresponding mesentery is generally recommended.

The presence of secondary lymphatic or hepatic dissemination is not considered as a contraindication to surgery, which should include the treatment of hepatic metastases [26].

Residual disease is managed through administration of chemotherapy associated with symptomatic inhibition therapy with octreotide acetate (Sandostatin® LAR; Novartis Pharmaceuticals Corporation, East Hanover, NJ, USA). Five-year survival ranges around 75% [5] for patients with bowel circumscribed disease, while for patients with lymphatic or hepatic involvement it decreases to 50% and 20%, respectively [27].

## Conclusions

Even though the strategy for adult patients of an incidental, asymptomatic and macroscopically harmless finding of MD during surgery performed for other reasons is still not codified, we recommend prophylactic excision in order to avoid any further risk.

## Consent

Written informed consent was obtained from the patient for publication of this case report and any accompanying images. A copy of the written consent is available for review by the Editor-in-Chief of this journal.

## Abbreviations

CT: Computed tomography; GIST: Gastrointestinal stromal tumor; H & E: Hematoxylin and eosin; MD: Meckel’s diverticulum; SEER: Surveillance, Epidemiology, and End Results; SMA: Superior mesenteric artery.

## Competing interests

None of the authors involved in manuscript preparation have any competing interests towards the manuscript itself, neither financial nor moral conflicts. None of the authors received support in the form of grants, equipment and/or pharmaceutical items.

## Authors’ contributions

All authors contributed equally to this work, read, and approved the final manuscript.
